# Water-induced degradation and small-strain dynamic characteristics of tremella fuciformis polysaccharide treated loess

**DOI:** 10.1371/journal.pone.0362891

**Published:** 2026-11-13

**Authors:** Shengting Shi, Haokun Tong, Jingxuan Zhou, Zhanpeng Ji, Xinlei Zhang

**Affiliations:** 1 School of Digital Creativity, Jiangsu Vocational College of Business, Nantong, China; 2 Research Center of Urban Underground Space, Nanjing Tech University, Nanjing, China; China University of Mining and Technology, CHINA

## Abstract

The susceptibility of loess to water-induced deterioration poses a major threat to geotechnical stability. This study investigates the small-strain dynamic behavior of loess treated with tremella fuciformis polysaccharide (TFP), a sustainable biopolymer, using resonant column tests. The evolution of the maximum shear modulus (*G*_max_) and reference shear strain (*γ*_r_) was systematically analyzed under varying applied confining pressures, water contents, and TFP dosages. Results show that for untreated loess, *G*_max_ follows a power-law growth with confining pressure and linear attenuation with water content. TFP treatment significantly bolsters the small-strain shear stiffness; at a 2% dosage (*σ*_c_ = 150 kPa, *w* = 13%), *G*_max_ increases from 63.12 MPa to 139.93 MPa. TFP treatment enhances the initial shear stiffness while altering the reference shear strain, indicating changes in the strain-dependent modulus reduction characteristics. A unified multi-factor model was developed to characterize the variations in *G*_max_ and *γ*_r_, providing a quantitative description of the strain-dependent modulus reduction behavior. SEM observations reveal film-like and filament-like features around soil particles, suggesting microstructural changes associated with TFP treatment. These findings highlight the potential of TFP as a sustainable bio-based approach to enhance the small-strain shear stiffness of loess, particularly under high-water-content conditions.

## Introduction

Loess, a typical Quaternary aeolian deposit, covers approximately 10% of the Earth’s land surface and is widely distributed across arid and semi-arid regions in Eurasia, North America, and South America. This material is characterized by high porosity, an under-consolidated state, and a metastable structure composed of soluble salts and clay particles [1–3]. While loess exhibits relatively high structural strength in its natural state, it faces severe stability challenges due to the dual impact of frequent extreme precipitation and tectonic activities in seismic zones under global climate change [4,5]. The coupling effect of moisture infiltration and dynamic loading (e.g., earthquakes and high-speed railway vibrations) often triggers irreversible and severe stiffness softening and skeleton collapse, leading to large-scale flowslides, ground settlement, and catastrophic infrastructure failure [6–8]. Therefore, exploring efficient reinforcement technologies that can simultaneously enhance dynamic stiffness and inhibit water-induced deterioration has become an urgent task in the field of global geological engineering.

For loess reinforcement, the industry has long relied on traditional inorganic stabilizers such as Portland cement, lime, or gypsum [9,10]. Despite their significant strength-enhancing effects, their production processes involve substantial carbon emissions and may lead to soil salinization and heavy metal contamination of groundwater. Driven by the global goals of “carbon neutrality” and “sustainable infrastructure,” biopolymer-based soil stabilization has emerged as a highly competitive green alternative. Liu et al. [11] employed calcium alginate (CA) to establish Ca^2+^ cementation between loess particles, which enhanced tensile performance through agglomeration and maintained structural stability under repetitive wetting-drying cycles. Albenga and Francisca [12] used natural biopolymers like xanthan gum and guar gum to establish gel-bonding, significantly improving the stiffness of compacted loess and confirming the technical feasibility of bio-cementation in collapsible ground improvement. Additionally, Du et al. [13] improved the water retention of loess through composite reinforcement with guar gum and basalt fibers and developed a predictive model considering environmental degradation. These studies provide important practical paradigms for the eco-friendly improvement of moisture-sensitive loess.

However, most existing studies have focused on the static mechanical strength and macro-durability of biopolymer-reinforced soils. For instance, Gu et al. [14] explored the static mechanical behavior and pore-filling mechanisms of reinforced soil under freeze-thaw cycles, while Liang et al. [15] quantitatively evaluated the static damage evolution of fiber-biopolymer reinforced loess during wetting-drying cycles. In contrast, there remains a significant research gap concerning the dynamic evolutionary laws and nonlinear attenuation characteristics of reinforced loess. First, the nonlinear evolutionary path of the dynamic properties of loess reinforced with tremella fuciformis polysaccharide (TFP) is not yet clearly understood. Second, a complex trade-off exists between moisture-induced stiffness degradation and polysaccharide-induced cementation reinforcement; currently, there is a lack of a unified predictive framework that effectively couples applied confining pressure, water content, and reinforcement dosage. Furthermore, the microstructural features associated with TFP reinforcement and their contributions to the dynamic shear stiffness of loess under different water contents remain insufficiently understood.

Crucially, biopolymers influence the strain-dependent nonlinear evolution in ways that traditional stabilizers do not. Unlike conventional inorganic binders that create rigid but brittle frameworks, TFP introduces a viscoelastic and flexible bonding phase into the soil-particle system. Tremella fuciformis polysaccharide (TFP) are high-molecular-weight polymers rich in hydroxyl and carboxyl groups, capable of forming high-tenacity coatings on mineral surfaces through hydrogen bonding and Van der Waals forces [16–18]. However, this bio-network-reconstructed dynamic response is highly susceptible to modulation by dosage gradients and ambient moisture, and its stability remains to be quantitatively defined.

To promote its application in seismic engineering, this study utilized resonant column tests (RCT) to conduct dynamic parameter testing across 27 experimental conditions with varying applied confining pressures, water contents, and TFP dosages. By analyzing the evolution of the maximum dynamic shear modulus (*G*_max_) and reference shear strain (*γ*_r_), a unified dynamic response predictive model considering multi-factor coupling was developed. Meanwhile, scanning electron microscopy (SEM) was employed to examine the microstructural features associated with TFP reinforcement and their possible contributions to the enhanced dynamic shear stiffness under different water contents. These findings provide experimental insights into the small-strain dynamic characteristics of TFP-treated loess and its potential application in sustainable ground improvement.

## Materials and methods

### Materials and soil characterization

The soil used in this study was collected from Baota District, Yan’an City, Shaanxi Province, China, located in the central part of the Chinese Loess Plateau, which is a typical loess hilly-gully region. To ensure stratigraphic representativeness and avoid the influence of the weathered surface layer and anthropogenic fills, the samples were obtained from a depth of 3.0–5.0 m, corresponding to the Late Pleistocene (Q3) Malan loess [19–21]. The sampling site was located in a publicly accessible, non-protected area and was not privately owned. No formal permits or regulatory approvals were required for the collection of ordinary loess samples at this location, and no protected species were involved.

The natural water content and density were 13.2% and 1.56 g/cm^3^, respectively. The liquid limit and plastic limit were 28.5% and 18.2%, yielding a plasticity index (*I*_P_) of 10.3. Standard Proctor tests indicated a maximum dry density is 1.76 g/cm^3^ and an optimum water content of 16.8%. Fig 1 presents the particle size distribution curve of the loess, where silt-sized particles (0.005–0.075 mm), clay-sized particles (<0.005 mm), and sand-sized particles (>0.075 mm) account for 61%, 36%, and 3%, respectively. Accordingly, the soil is classified as clayey silt loess [22,23]. This type of loess is characterized by a metastable structure and high collapsibility, which are critical to its dynamic response and water-induced degradation behavior.

**Fig 1:**
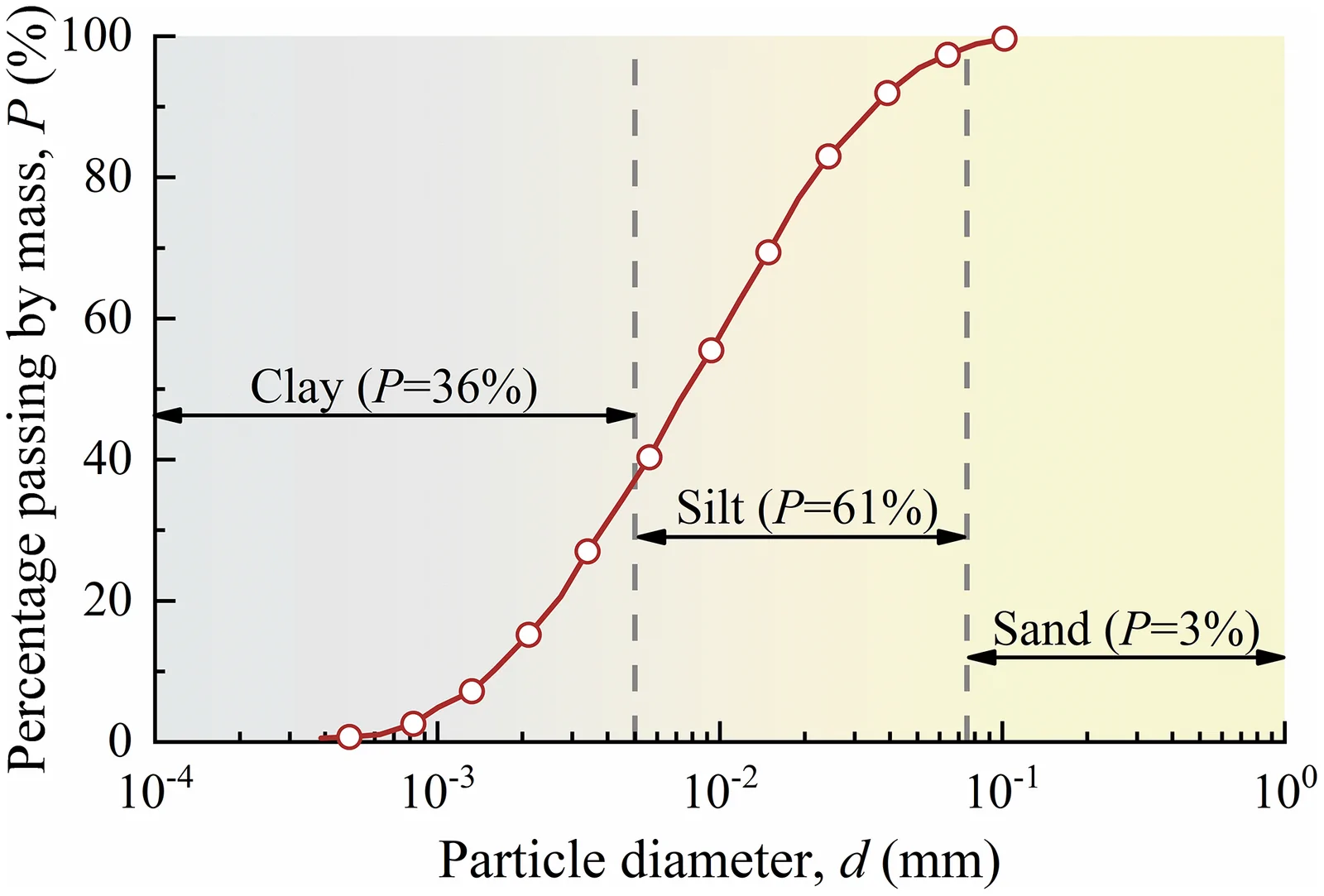
Grain-size distribution curve of loess.

### Tremella fuciformis polysaccharide

Tremella fuciformis polysaccharide (TFP), extracted from the fruiting body of Tremella fuciformis, was selected as a representative fungal-derived biopolymer. Due to its strong adhesion, film-forming ability, and biochemical stability [24,25], these characteristics suggest that TFP has the potential to enhance the dynamic shear stiffness of loess under varying water contents.

The standardized extraction procedure of TFP (Fig 2) mainly consists of three stages: pretreatment, extraction, and purification. (a) The dried fruiting bodies were ground and passed through a 40-mesh (0.425 mm) sieve, followed by immersion in 95% ethanol at a solid–liquid ratio of 1:10 for 12 h to remove lipid-soluble impurities and pigments. (b) The treated powder was then extracted in distilled water at 100 °C for 2 h. The suspension was centrifuged at 10,000 rpm for 10 min to obtain the supernatant, which was subsequently concentrated to one-quarter of its original volume using a rotary evaporator at 50 °C under reduced pressure. (c) Three volumes of 95% ethanol were added to the concentrated solution and kept for 12 h to induce polysaccharide precipitation. The precipitate was further collected by centrifugation and freeze-dried under vacuum to obtain the final white powder product. This extraction protocol ensures that TFP possesses a high density of active hydroxyl groups and a long-chain molecular structure, which are essential for interparticle bonding and structural stabilization in treated soils [26–28]. Based on this, TFP-treated loess specimens with different biopolymer contents were prepared for subsequent dynamic testing.

**Fig 2:**
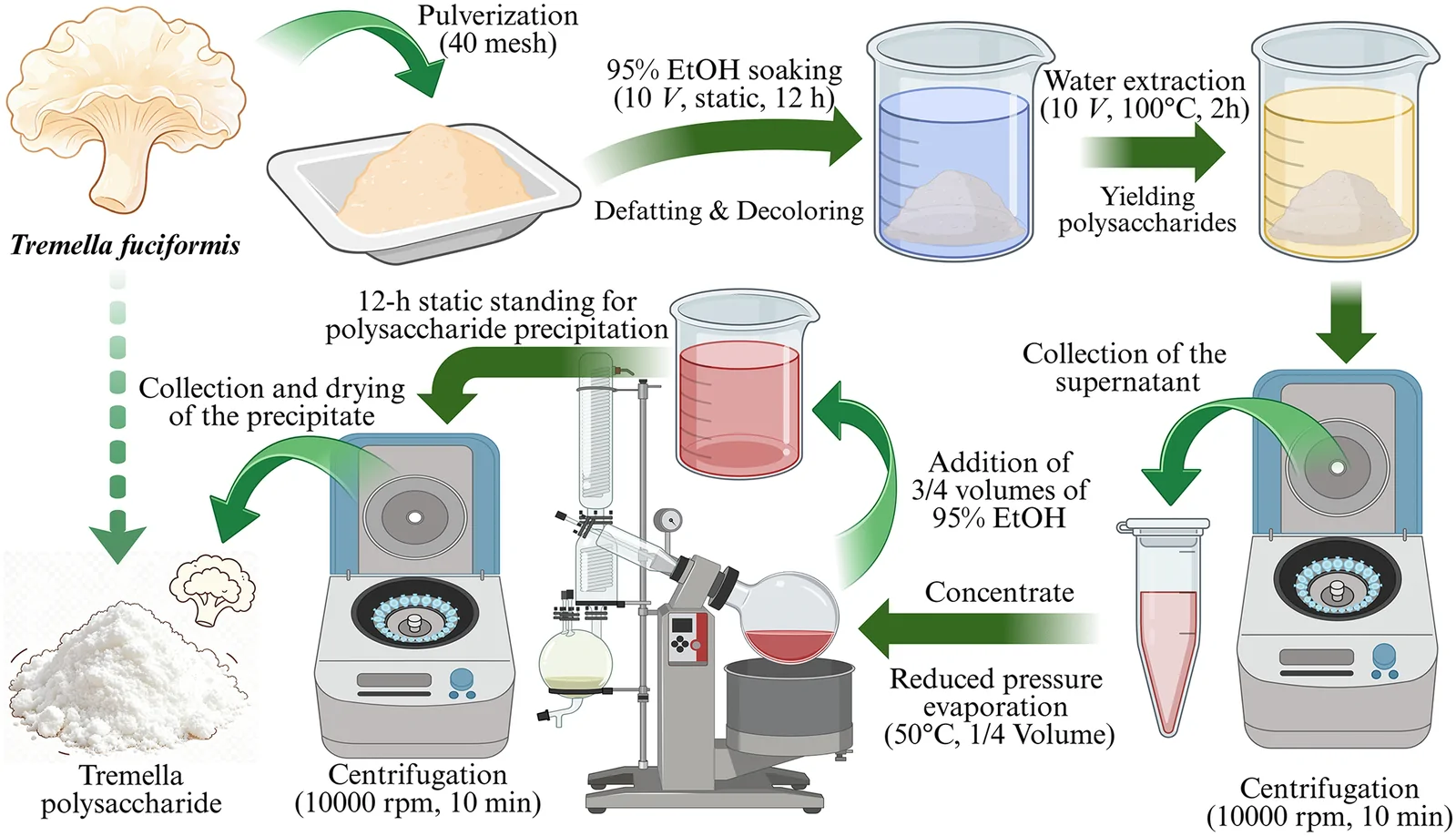
Schematic flowchart of the extraction and purification process of TFP. Created in BioRender. Ji, Z. (2026) https://BioRender.com/aov2s7n. Note: *V* represents the initial volume of the *Tremella fuciformis* fruiting bodies.

### Specimen preparation

To address the pronounced water-induced deterioration characteristics of loess and the associated volumetric instability during moisture conditioning [29–31], a specimen preparation protocol consisting of large-scale preforming, controlled moisture equilibration, and post-trimming was developed (Fig 3).

**Fig 3:**
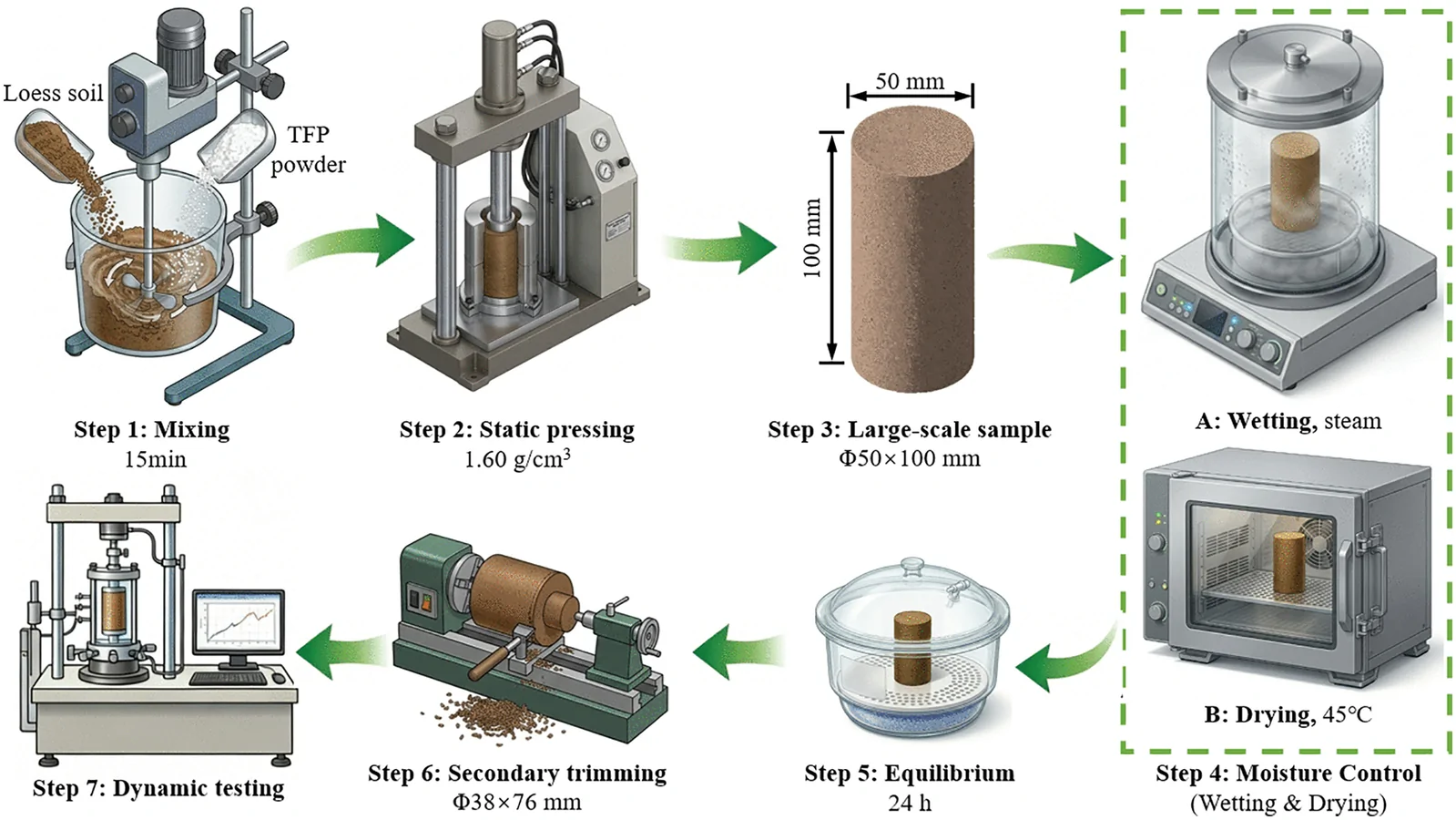
Schematic illustration of the sample preparation and moisture regulation protocol for TFP-treated loess. The individual panels were generated with the assistance of AI and arranged and formatted by the authors.

First, oven-dried loess was thoroughly mixed with TFP powder at predetermined mass fractions using a mechanical mixer for 15 min to ensure material homogeneity (Step 1). The mixtures were then statically compacted to a target initial dry density of 1.60 g/cm^3^ into cylindrical preformed specimens with a diameter of 50 mm and a height of 100 mm (Steps 2 and 3).

A consistent target initial dry density was adopted to establish comparable initial compaction conditions among specimens, following the controlled-variable approach used in previous experimental investigations of loess [32,33]. The use of oversized preformed specimens was intended to reserve sufficient trimming allowance to accommodate volumetric deformation induced during subsequent moisture conditioning. It should be noted that the final dry density after moisture conditioning and trimming was not independently measured. Therefore, the experimental results are interpreted as the dynamic response associated with different moisture states under a consistent target initial dry density, rather than the isolated effect of water content at an identical final dry density.

Moisture regulation (Step 4) involved two pathways: humidification and dehydration. During humidification, a non-contact vapor diffusion method was employed, in which specimens were placed above a heated system equipped with a 0.075 mm perforated steam rack. Uniform moisture increase was achieved through gentle vapor diffusion. The process was initiated upon vapor release, and specimen mass was continuously monitored; if the target water content was not reached, measurements were taken at 30 min intervals thereafter. For dehydration, specimens were dried in a forced-air oven at 45 °C, with mass monitored to ensure a gradual and controlled reduction in water content.

After reaching the target water content, specimens were sealed in airtight containers and allowed to equilibrate for 24 h to eliminate internal temperature and moisture gradients and to stabilize volumetric strain (Step 5). Subsequently, the equilibrated preformed specimens were precision-trimmed using a high-accuracy cutting device to final dimensions of 38 mm in diameter and 76 mm in height (Step 6).

This preparation method effectively removes the disturbed outer layer and minimizes the effects of inevitable volumetric expansion–contraction and boundary disturbances during moisture conditioning. As a result, it significantly improves specimen regularity and structural uniformity, providing a reliable basis for subsequent high-precision dynamic testing (Step 7).

### Testing apparatus and experimental program

#### Resonant column tests.

A GCTS TSH-100 resonant-column system was employed to investigate the strain-dependent dynamic shear modulus of TFP-treated loess over a shear strain range of approximately 10^-6^-10^-2^ [34]. The apparatus adopts a fixed-base configuration with top torsional excitation generated by an electromagnetic driving system. Before testing, a standard aluminum calibration specimen was used to check the system response and relevant apparatus parameters. Preliminary frequency sweeps were conducted to verify the stability of the resonant response. The dynamic shear modulus was determined from the measured resonant response based on torsional wave propagation theory [35].

All specimens were subjected to isotropic consolidation under the designated confining pressure after installation. Consolidation was considered complete when the rate of specimen height change was less than 0.01 mm/h, ensuring a stable stress state prior to dynamic loading. Following consolidation, sinusoidal torsional excitation was applied at different excitation levels. Frequency sweeps were performed at each excitation level, and the resonant frequency was identified from the peak response amplitude. The corresponding shear strain and dynamic shear modulus were determined from the measured resonant response. To systematically evaluate the effects of key variables on the dynamic stiffness of the treated loess, the experimental program was designed as summarized in Table 1. Four TFP content levels (*C* = 0.5%, 1.0%, 1.5%, and 2.0%) were considered, along with untreated loess specimens (*C* = 0%) as a control group to quantify the improvement effect of TFP. The testing matrix also included five water content levels (*w* = 5.0%, 9.0%, 13.0%, 17.0%, and 20.0%) and five applied confining pressures (*σ*_c_ = 50, 100, 150, 200, and 300 kPa). Here, *σ*_c_ denotes the externally applied isotropic confining pressure.

**Table 1:** Resonant column test condition.

Test ID	*C* (%)	*w* (%)	*σ*_c_ (kPa)	*G*_max_ (MPa)	*γ*_r_ (%)
C0-w5-s150	0	5	150	103.84	0.039
C0-w9-s150	0	9	150	81.61	0.072
C0-w13-s150	0	13	150	63.12	0.118
C0-w17-s150	0	17	150	52.88	0.196
C0-w20-s150	0	20	150	38.29	0.275
C0-w13-s50	0	13	50	45.99	0.041
C0-w13-s100	0	13	100	61.22	0.081
C0-w13-s200	0	13	200	81.34	0.152
C0-w13-s300	0	13	300	97.71	0.226
C0-w5-s50	0	5	50	63.68	0.015
C0-w20-s50	0	20	50	23.06	0.110
C0-w5-s300	0	5	300	142.61	0.075
C0-w20-s300	0	20	300	51.74	0.559
C0.5-w13-s150	0.5	13	150	87.24	0.115
C1.0-w13-s150	1.0	13	150	104.88	0.095
C1.5-w13-s150	1.5	13	150	119.03	0.075
C2.0-w13-s150	2.0	13	150	139.93	0.044
C1.0-w5-s150	1.0	5	150	166.19	0.035
C1.0-w9-s150	1.0	9	150	134.89	0.055
C1.0-w17-s150	1.0	17	150	75.89	0.170
C1.0-w20-s150	1.0	20	150	58.04	0.240
C1.0-w13-s300	1.0	13	300	138.97	0.183
C2.0-w13-s300	2.0	13	300	181.34	0.086
C2.0-w5-s150	2.0	5	150	224.67	0.015
C2.0-w20-s150	2.0	20	150	74.38	0.110
C1.0-w13-s50	1.0	13	50	74.21	0.036
C2.0-w13-s50	2.0	13	50	100.92	0.016

Notes: *C*, *w*, and *σ*_c_ denote TFP content (%), water content (%), and applied confining pressure (kPa), respectively. Test IDs are formatted as “C[content]-w[water]-s[confining pressure].

#### Scanning electron microscopy (SEM) analysis.

Scanning electron microscopy (SEM) was employed to examine the microstructural features of TFP-treated loess under representative conditions and explore their possible contributions to the observed enhancement in dynamic shear stiffness. To ensure the representativeness of microstructural observations while minimizing disturbance to the soil skeleton during sample preparation, small blocks with dimensions of 10 mm (length) × 10 mm (width) × 20 mm (height) were carefully extracted from the central region of specimens after dynamic testing. To reduce morphological variability induced by different observation orientations, all observation surfaces were taken from vertical cross-sections, and the cutting and placement directions were kept strictly consistent for all samples. The samples were air-dried for 72 h and then manually fractured to obtain fresh surfaces with minimal mechanical disturbance. Subsequently, the specimens were gold-coated to enhance electrical conductivity prior to SEM imaging. These observations were used to characterize the microstructural features associated with TFP treatment and explore their possible contributions to the observed changes in dynamic shear stiffness.

## Dynamic response and predictive modeling of untreated loess

### Nonlinear decay of dynamic shear modulus and its sensitivity

Fig 4 illustrates the typical nonlinear decay of dynamic shear modulus (*G*) with increasing shear strain (*γ*) for untreated loess, highlighting its pronounced sensitivity to applied confining pressure (*σ*_c_) and water content (*w*). At a constant water content (*w* = 13%), the *G*-*γ* curves exhibit a systematic upward shift as *σ*_c_ increases from 50 to 300 kPa (Fig 4a). This response is attributed to the enhancement of interparticle contact stress and mechanical interlocking under higher applied confining pressure, which leads to an increase in the initial shear stiffness of the soil skeleton [36,37]. In contrast, Fig 4b illustrates the reduction in dynamic shear modulus with increasing water content at a constant applied confining pressure (*σ*_c_ = 150 kPa). For unsaturated loess, variations in water content may affect matric suction, interparticle contact conditions, and water-sensitive bonding within the soil skeleton. These factors may collectively contribute to the observed decrease in *G* [38].

**Fig 4:**
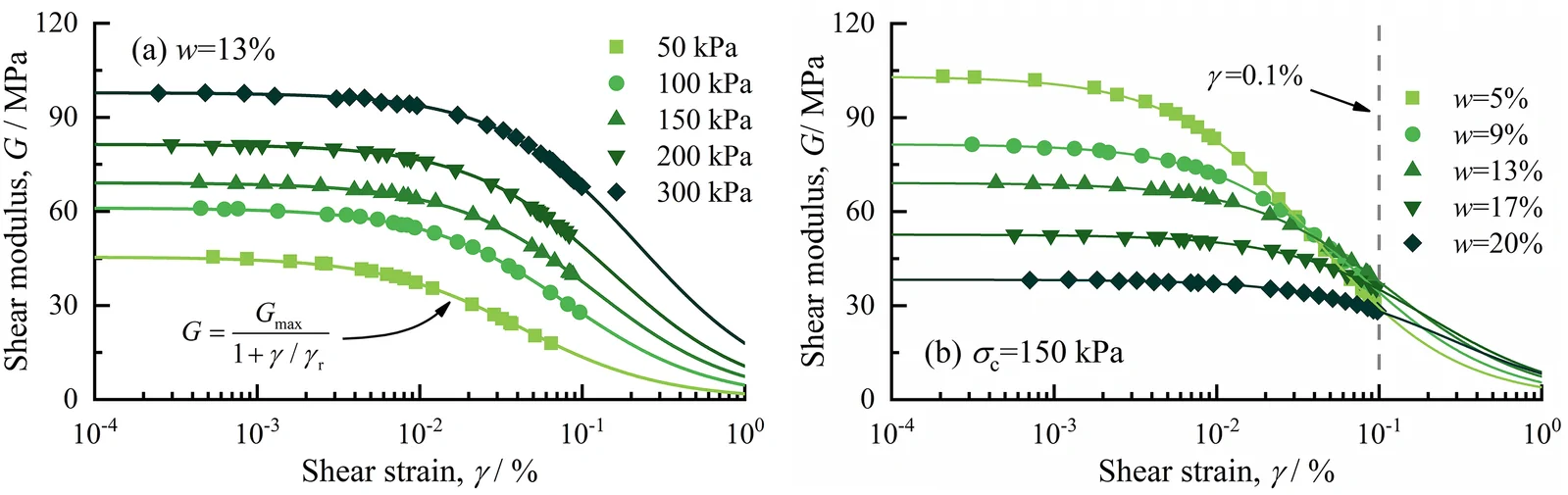
Effect of *σ*_c_ and *w* on the dynamic shear modulus of untreated loess: (a) different applied confining pressures (*w* = 13%); (b) different water contents (*σ*_c_ = 150 kPa).

Notably, the maximum dynamic shear modulus (*G*_max_) demonstrates a higher sensitivity to moisture fluctuations compared to the medium-to-high strain stages. Furthermore, the contribution of water content to dynamic stiffness exhibits a distinct strain-threshold behavior. At small strains (*γ* < 10^−2^%), soil stiffness may be strongly influenced by the initial soil fabric and matric suction; however, as the shear strain approaches 0.1%, the decay curves for varying water contents tend to converge. This phenomenon reveals an evolution of loess dynamic behavior from a structure-controlled to a stress-controlled regime, where the original skeleton stability and suction-induced bonds undergo irreversible degradation with increasing particle displacement, thereby diminishing the structural advantages provided by lower initial water content.

To quantitatively characterize the nonlinear evolution of the dynamic properties of unreinforced loess, the classic Hardin model [39,40] was employed to perform nonlinear regression analysis on the measured *G*-*γ* curves. The model is expressed as follows:


$$\begin{array}{c} G=\frac{G_{max}}{1+\gamma /\gamma_{\text{r}}} \end{array}$$ (1)

where *G*_max_ (MPa) represents the maximum dynamic shear modulus, denoting the initial shear stiffness of the soil at small strain levels; *γ*_r_ is the reference shear strain, defined as the strain value at which the modulus ratio *G*/*G*_max_ reduces to 0.5. This parameter characterizes the evolution rate of the soil’s dynamic nonlinearity, with a larger *γ*_r_ indicating a more gradual attenuation of stiffness with increasing shear strain. Table 1 summarizes the fitted values of *G*_max_ and *γ*_r_ across various experimental conditions. The results demonstrate that the model satisfactorily captures the mechanical response during the transition from the small-strain linear regime to the large-strain nonlinear regime.

### Evolution of *G*_max_ and *γ*_r_ with *σ*_c_ and *w* for untreated loess

As a primary indicator of the initial dynamic stiffness of the soil skeleton, the evolution of the maximum dynamic shear modulus (*G*_max_) is essentially governed by the dual regulation of the strengthening effect of applied confining pressure (*σ*_c_) and the deterioration effect of water content (*w*) (Fig 5). As shown in Fig 5a, *G*_max_ exhibits a nonlinear growth trend with increasing *σ*_c_ across various water contents, which can be described by the following power-law relationship:

**Fig 5:**
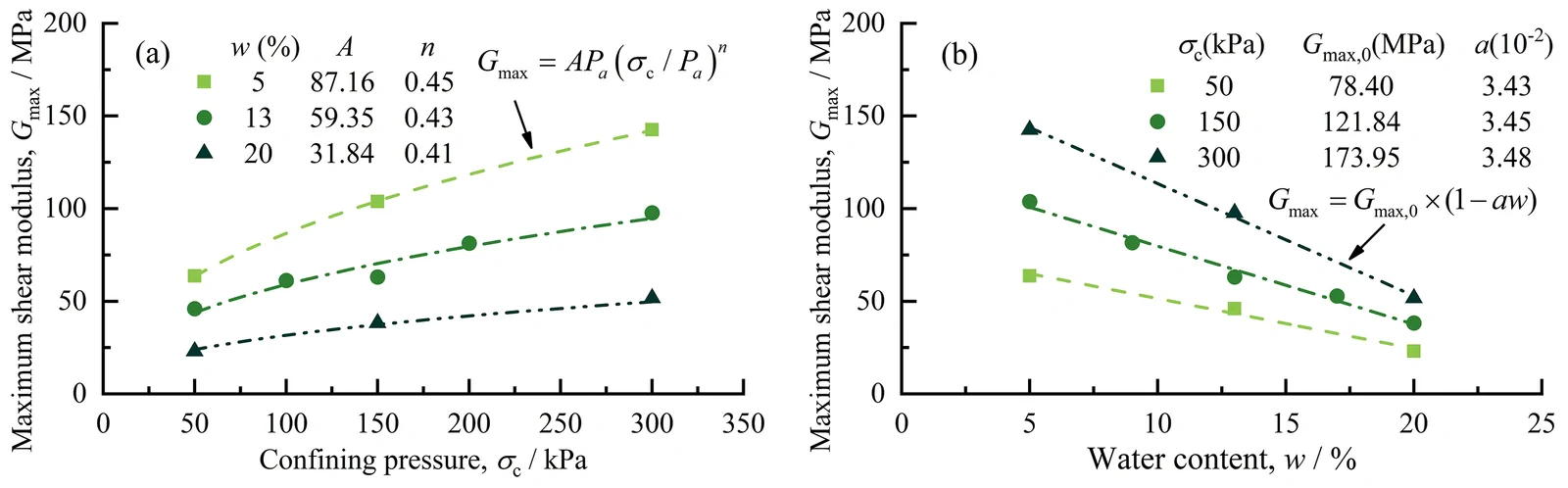
Evolution of *G*_max_ with applied confining pressure and water content: (a) different applied confining pressures; (b) different water contents.


$$\begin{array}{c} G_{max}=AP_{a}{\left(\sigma_{\text{c}}/P_{a}\right)}^{n} \end{array}$$ (2)

where *A* is a dimensionless parameter reflecting the initial physical state and structural fabric of the soil; *n* is the modulus exponent characterizing the stress-dependency of the dynamic shear modulus; and *P*_*a*_ is the reference atmospheric pressure (101.3 kPa).

From a micromechanical perspective, an increase in confining pressure enhances skeleton density by compressing pore volumes, which elevates the number of particle contacts per unit volume and strengthens frictional interlocking at the contact interfaces. Regression results indicate that the modulus exponent n increases significantly with decreasing water content. This reveals a heightened stress sensitivity of the untreated loess skeleton under low-moisture conditions, suggesting that a drier skeleton more effectively translates macroscopic external stress into microscopic contact stiffness.

Fig 5b illustrates the approximately linear decrease in *G*_max_ with increasing water content under different applied confining pressures. For unsaturated loess, this response may reflect the combined effects of variations in matric suction, interparticle contact conditions, and water-sensitive bonding. The individual contributions of these factors cannot be quantified without independent suction measurements. Furthermore, although the absolute reduction in *G*_max_ increases with applied confining pressure, the fitted decay factor *a* exhibits only slight variation, indicating broadly consistent normalized moisture-dependent attenuation characteristics across the investigated confining pressures.

Fig 6 illustrates that the reference shear strain (*γ*_r_) exhibits a significant monotonic increase with both applied confining pressure (*σ*_c_) and water content (*w*), reflecting that the nonlinear evolutionary path of soil dynamics is highly modulated by environmental state variables. As shown in Fig 6a, *γ*_r_ demonstrates a strong linear correlation with *σ*_c_ at a constant water content, which can be expressed as:

**Fig 6:**
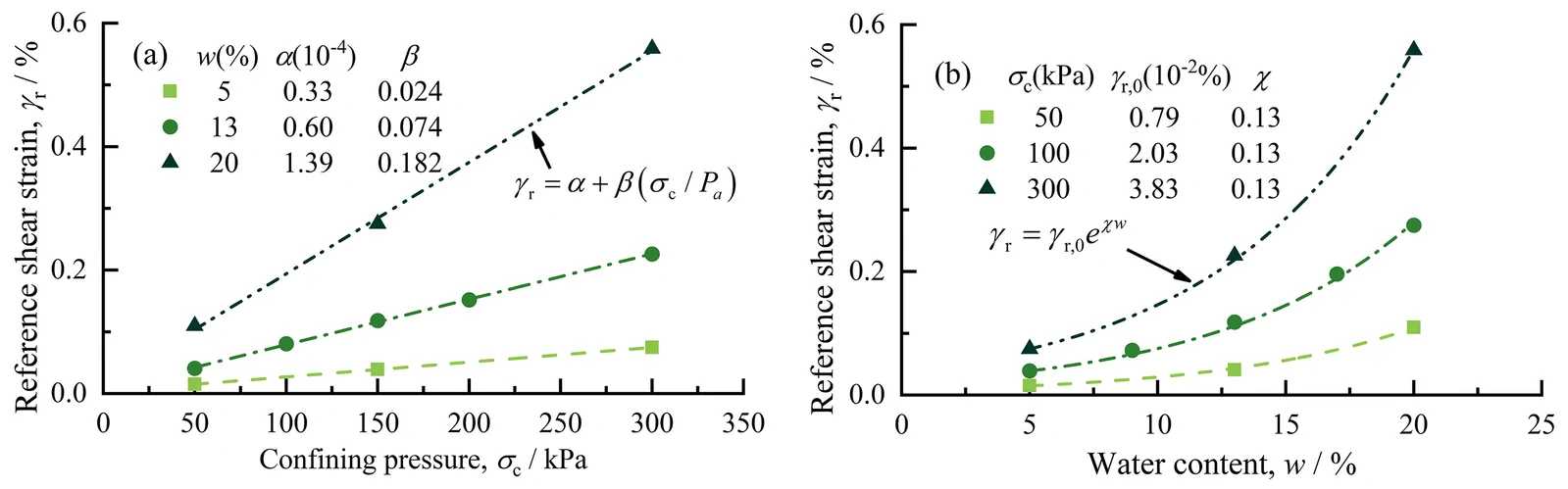
Evolution of *γ*_r_ with applied confining pressure and water content: (a) different applied confining pressures; (b) different water contents.


$$\begin{array}{c} \gamma_{\text{r}}=\alpha +\beta \times \left(\sigma_{\text{c}}/P_{a}\right) \end{array}$$ (3)

This is attributed to the fact that elevated confining pressure enhances the interlocking forces and normal contact stresses between loess particles, thereby bolstering the deformation resistance of the skeleton structure. Macroscopically, this strengthening effect is manifested as a rightward shift of the modulus attenuation curves toward larger strain levels. In other words, high confining pressure delays the transition of the soil into the stage of severe nonlinear evolution, effectively broadening its linear elastic response regime.

In contrast to the linear strengthening effect of confining pressure, Fig 6b reveals an exponential growth pattern of *γ*_r_ with respect to water content. The fitting model is as follows:


$$\begin{array}{c} \gamma_{\text{r}}=\gamma_{\text{r,0}}\times e^{\chi w} \end{array}$$ (4)

The increase in *γ*_r_ with water content indicates a more gradual normalized shear modulus reduction as shear strain increases. For unsaturated loess, this response may be associated with moisture-induced changes in interparticle contacts and water-sensitive bonding. The fitted exponential coefficient *χ* exhibits limited variation across the investigated confining pressures, suggesting broadly consistent water-dependent evolution of *γ*_r_ under different stress conditions.

### Model formulation and assessment of dynamic shear modulus

The preceding analysis shows that *G*_max_ increases with applied confining pressure following a power-law relationship and decreases approximately linearly with water content. Although the normalized water-induced stiffness attenuation exhibits limited stress dependence, the absolute stiffness reduction increases with confining pressure because of the higher baseline stiffness. Accordingly, an integrated model for *G*_max_ is established as follows:


$$\begin{array}{c} G_{max}=AP_{a}{\left(\sigma_{\text{c}}/P_{a}\right)}^{n}\left(1-aw\right) \end{array}$$ (5)

In this model, water content is incorporated as a linear deterioration factor. Specifically, the term *AP*_*a*_(*σ*_*c*_/*P*_*a*_)^*n*^ characterizes the stress-induced strengthening of the soil skeleton in a dry state, while (1 − *aw*) quantifies the fractional reduction in stiffness across varying water content levels.

Regarding the reference shear strain *γ*_r_, experimental data indicate that it increases linearly with confining pressure and escalates exponentially with water content. Considering the stress-independent nature of moisture modulation on *γ*_r_, the prediction model is constructed as follows:


$$\begin{array}{c} \gamma_{\text{r}}=\left(\alpha +\beta \times \left(\sigma_{\text{c}}/P_{a}\right)\right)e^{\chi w} \end{array}$$ (6)

where the bracketed term reflects the linear expansion of the quasi-elastic response regime by applied confining pressure, and the exponential term captures the plasticization process as the soil transitions toward a soft-plastic state induced by hydration.

The fitting performance of the proposed models was evaluated by comparing the calculated dynamic parameters with the experimental results (Fig 7). The calculated *G*_max_ and *γ*_r_ values are generally distributed close to the 1:1 identity line, indicating that the proposed functional relationships adequately describe the observed variations in dynamic shear characteristics. Additionally, the experimental data reported by Song et al. [36] were examined using the same functional forms, with the model parameters recalibrated for the corresponding dataset (Table 2). This comparison provides an assessment of the applicability of the proposed functional relationships to another loess material.

**Table 2:** Summary of empirical parameters for *G*_max_ and *γ*_r_ prediction models.

	*A*	*n*	*a*	*α*	*β*	*χ*
Present study	1.04	0.45	0.035	1.14 × 10^−3^	0.014	0.13
Song et al. [36]	0.62	0.60	0.013	0.042	0.033	−0.01

**Fig 7:**
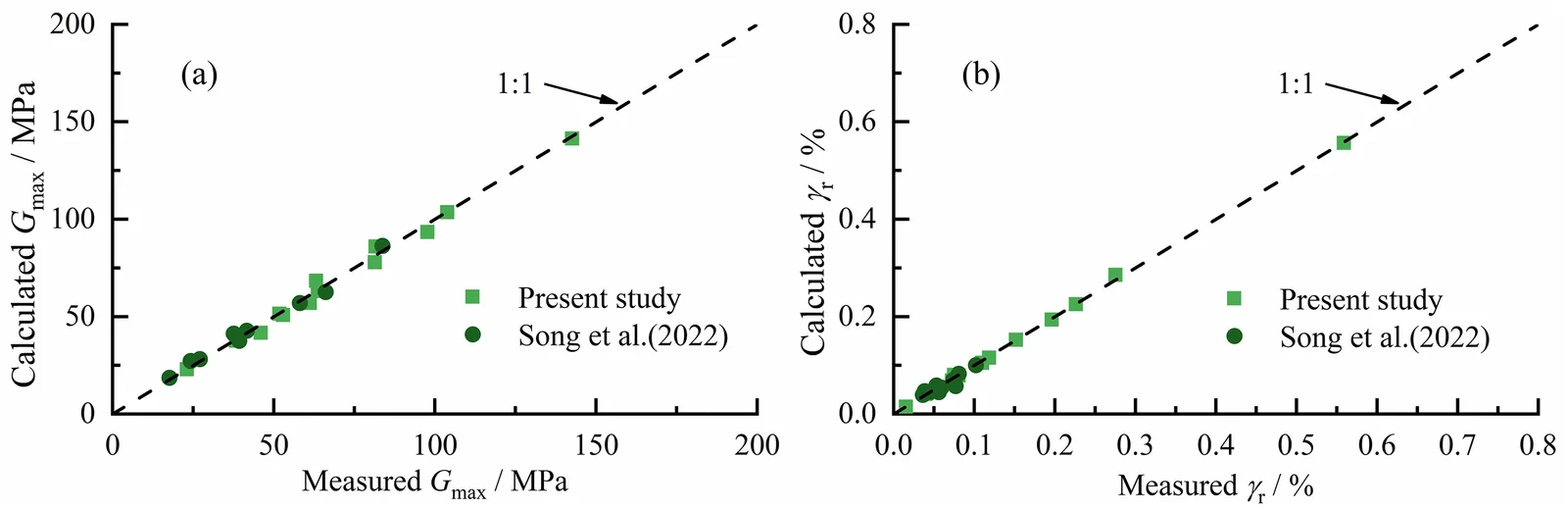
Comparison between the measured and calculated dynamic parameters: (a) maximum dynamic shear modulus *G*_max_; (b) reference shear strain *γ*_r_.

By substituting Eq. and into Eq., the unified predictive framework for the dynamic shear modulus is derived:


$$\begin{array}{c} G=\frac{AP_{a}{\left(\sigma_{\text{c}}/P_{a}\right)}^{n}\left(1-aw\right)}{1+\gamma /\left[\left(\alpha +\beta \times \left(\sigma_{\text{c}}/P_{a}\right)\right)e^{\chi w}\right]} \end{array}$$ (7)

This model provides a continuous mathematical description of the entire evolutionary process of loess dynamic characteristics. Its physical significance is well-defined: the numerator, combining a power-law function with a linear decay factor, represents the competitive regulation of the initial stiffness by stress-induced strengthening and water-induced deterioration. Meanwhile, the rectified term in the denominator accurately characterizes the modulation of environmental factors on the modulus attenuation rate and the plasticization process of the soil.

The applicability of the proposed functional relationships was further examined using the experimental data reported by Song et al. [36]. Considering the differences in material properties and experimental conditions, the model parameters were recalibrated for the corresponding dataset (Table 2). As shown in Fig 8, the recalibrated model reproduces the principal characteristics of the measured *G*-*γ* curves under different confining pressures and water contents, including the variations in initial stiffness and strain-dependent modulus reduction. These results indicate that the proposed functional relationships are applicable to another loess dataset, supporting their potential use in describing the dynamic shear response of loess with material-specific parameter calibration.

**Fig 8:**
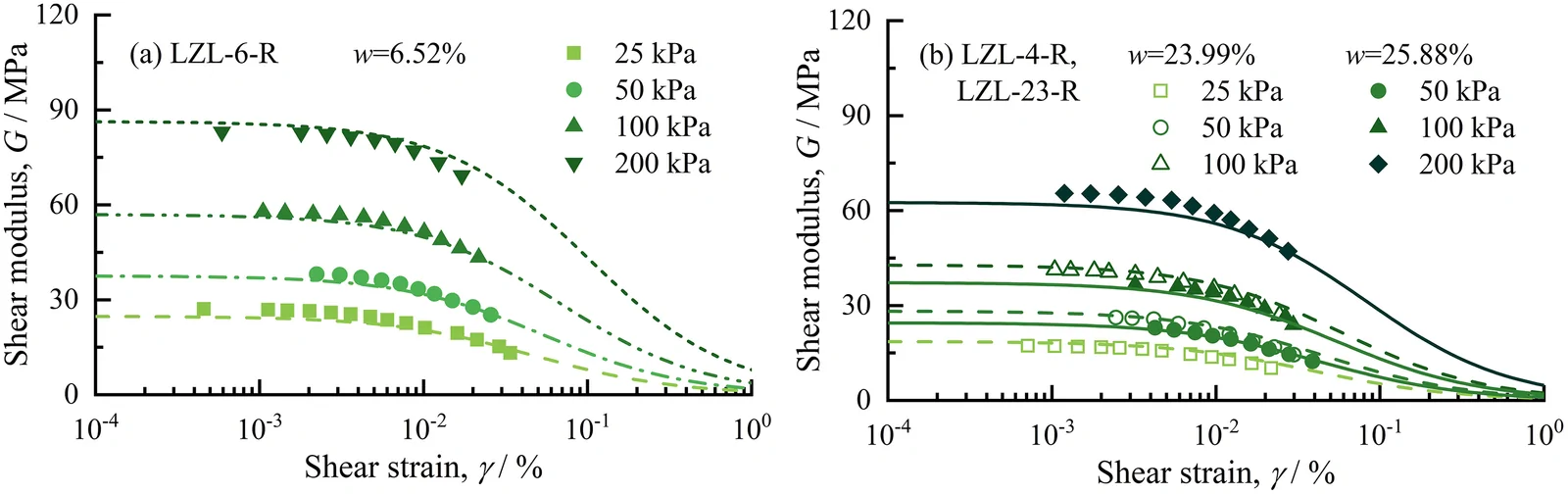
Comparison between measured and calculated dynamic shear modulus reduction curves using the data of Song et al. [36]: (a) low water content; (b) high water content.

## Dynamic response and revised model of TFP-treated loess

### Effects of TFP on shear modulus attenuation and water-induced degradation

Fig 9 illustrates the attenuation patterns of the dynamic shear modulus (*G*) for TFP-treated loess and its response mechanism to water-induced deterioration. A significant upward shift in *G* is observed with increasing TFP content (*C*) (Fig 9a). At a dosage of *C* = 2% (*σ*_c_ = 150 kPa, *w* = 13%), *G*_max_ reaches 139.93 MPa compared to 63.12 MPa for untreated loess. This marked stiffness enhancement may be associated with modifications to particle surface morphology and interparticle connections induced by TFP treatment.

**Fig 9:**
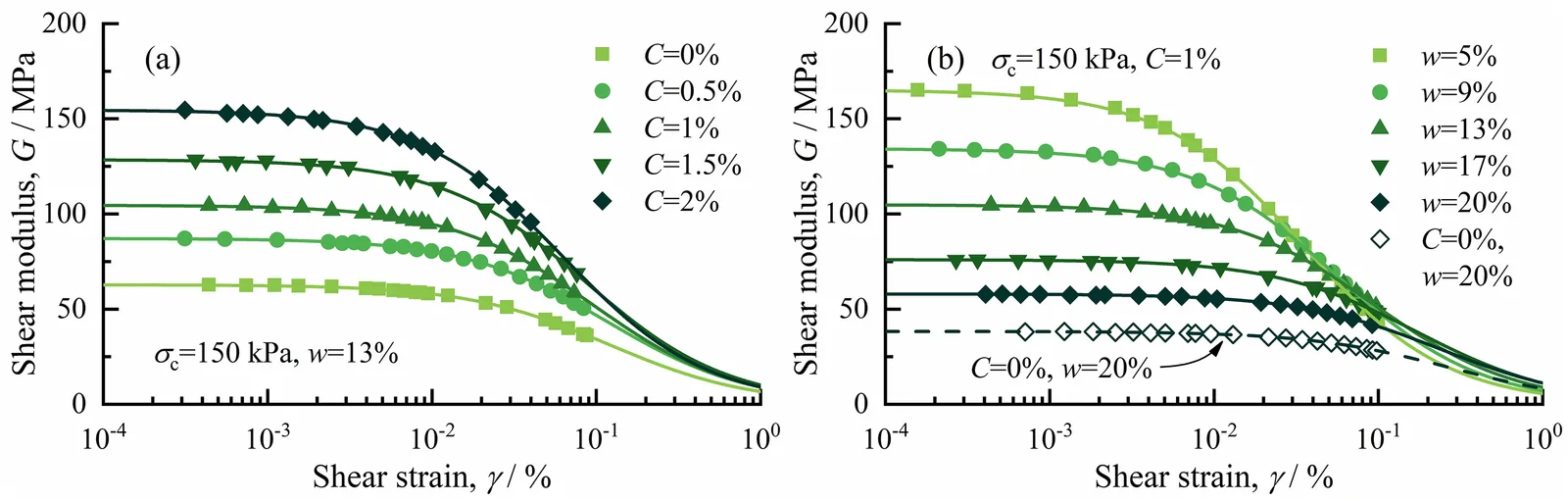
Dynamic shear modulus reduction curves of TFP-treated loess: (a) effect of TFP content; (b) effect of water content.

As water content (*w*) increases, the dynamic shear modulus (*G*) decreases for both untreated and TFP-treated loess (Fig 9b). However, at the same high water content (*w* = 20%), the *G*-*γ* curves of TFP-treated loess remain above those of untreated loess, reflecting the enhancement of absolute dynamic shear stiffness. This upward shift in the *G*-*γ* relationship indicates that TFP treatment maintains a higher stiffness level under wet conditions. Meanwhile, the more pronounced strain-dependent modulus reduction observed in specimens with higher TFP content highlights the influence of polysaccharide reinforcement on the nonlinear dynamic response of loess.

### Effect of TFP content on maximum dynamic shear modulus

Fig 10a illustrates the variation of *G*_max_ with TFP content (*C*) under various applied confining pressures. A strong positive linear correlation is observed between *G*_max_ and *C*. As the TFP concentration increases, the initial dynamic stiffness of the soil improves linearly, with the fitting slope *k*_*G*_ increasing significantly alongside the confining pressure. This indicates that the reinforcement efficiency of the polysaccharide is markedly stress-dependent: higher confining pressures facilitate tighter particle-to-particle contact, allowing the cementation structures formed by TFP molecules to operate on a more stable skeleton. This results in a synergistic “confining-reinforcement” enhancement effect.

**Fig 10:**
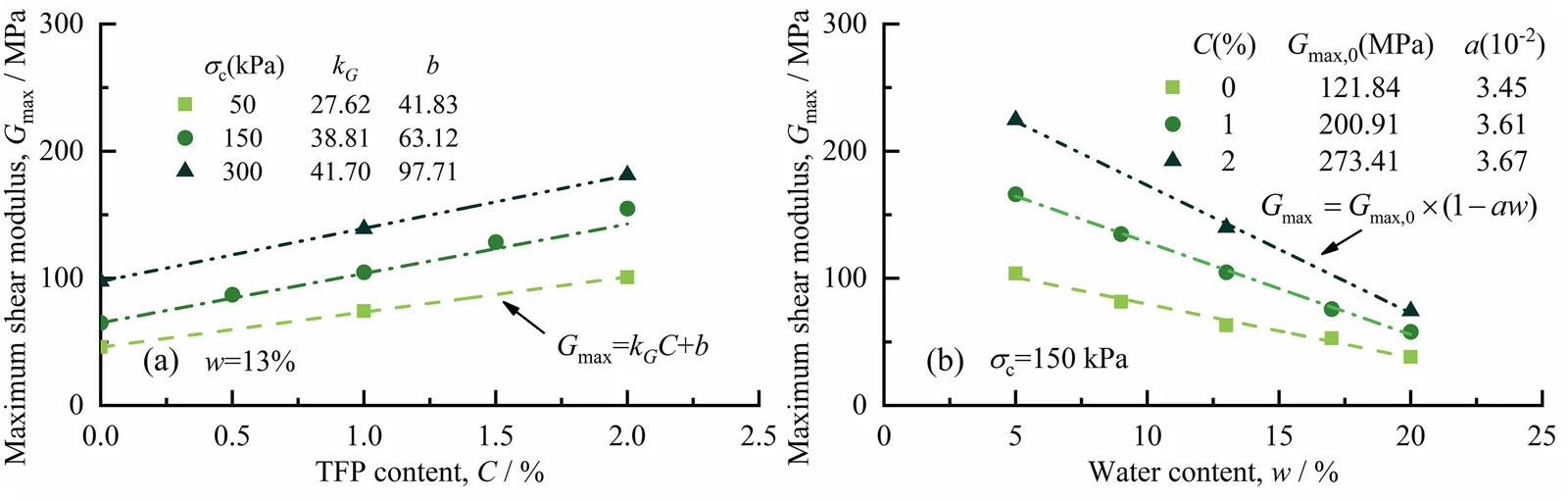
Evolution of *G*_max_ of TFP-treated loess: (a) effects of *C* and *σ*_*c*_; (b) effects of *w* and *C.*

The influence of water content on *G*_max_ of TFP-treated loess is shown in Fig 10b. For the TFP contents considered in Fig 10b, *G*_max_ exhibits an approximately linear decrease with increasing water content, consistent with the response observed in untreated loess. As the TFP content increases from 0% to 2%, the fitted parameter *G*_max,0_ increases from 121.84 MPa for untreated loess to 273.41 MPa for TFP-treated loess, whereas the decay factor a increases slightly from 0.0345 to 0.0367. The substantial increase in *G*_max,0_ indicates that TFP treatment enhances the overall stiffness level, while the limited variation in a reflects broadly consistent normalized moisture-dependent attenuation characteristics across different TFP contents. Consequently, TFP-treated loess maintains higher absolute stiffness under identical high-water-content conditions. For example, at a confining pressure of 150 kPa and a water content of 20%, *G*_max_ increases from 38.29 MPa for untreated loess to 74.38 MPa for 2% TFP-treated loess. These results demonstrate that the improvement in dynamic shear stiffness is primarily associated with an elevated stiffness level, accompanied by relatively limited changes in the normalized moisture-dependent attenuation behavior.

### Influence of TFP content on the reference shear strain

Fig 11a illustrates the variation of *γ*_r_ with TFP content (*C*) under different confining pressures. The *γ*_r_ generally decreases with increasing TFP content over the investigated conditions, although some nonmonotonic variations are observed at relatively low dosages. According to the Hardin model, a smaller *γ*_r_ corresponds to a more rapid reduction in normalized shear modulus with increasing shear strain. Fig 11b presents the influence of water content on *γ*_r_ at different TFP contents. *γ*_r_ increases with water content, indicating a more gradual normalized modulus reduction at higher water contents. The fitted exponential parameter *χ* remains approximately constant at 0.13 across the treated groups, suggesting similar water-dependent evolution characteristics. Overall, the observed variations in *G*_max_ and *γ*_r_ demonstrate that TFP reinforcement influences both the stiffness level and the characteristic strain scale of the nonlinear dynamic response.

**Fig 11:**
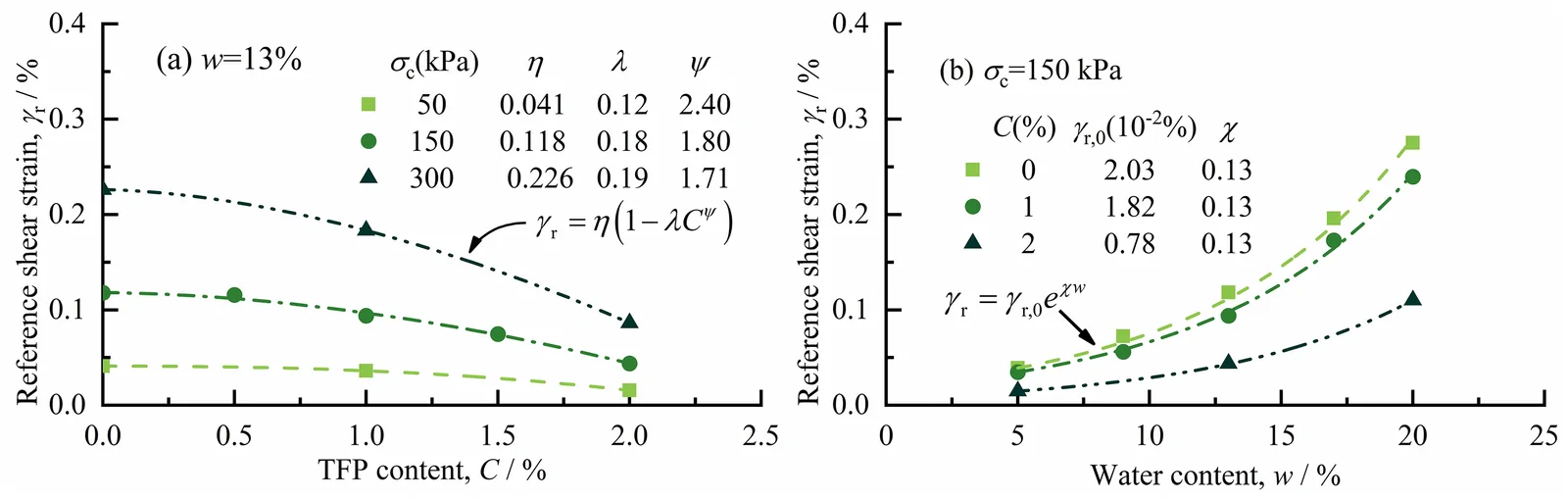
Evolution of *γ*_r_ of TFP-treated loess: (a) effects of *C* and *σ*_c_; (b) effects of *w* and *C.*

### Modified models for *G*_max_ and *γ*_r_ considering TFP content

To quantitatively characterize the reinforcement mechanism of TFP on the dynamic properties of loess, the relative growth rate (∆*G*_max_) and the relative change rate (∆*γ*_r_) were introduced to quantify the parameter deviation of the treated loess relative to the untreated loess. Fig 12a shows that ∆*G*_max_ exhibits a significant linear growth relationship with the TFP content (*C*), with a slope of 0.53. Based on this observation, a TFP enhancement factor (1 + *kC*) was integrated into the previously developed model to construct a comprehensive revised model for *G*_max_:

**Fig 12:**
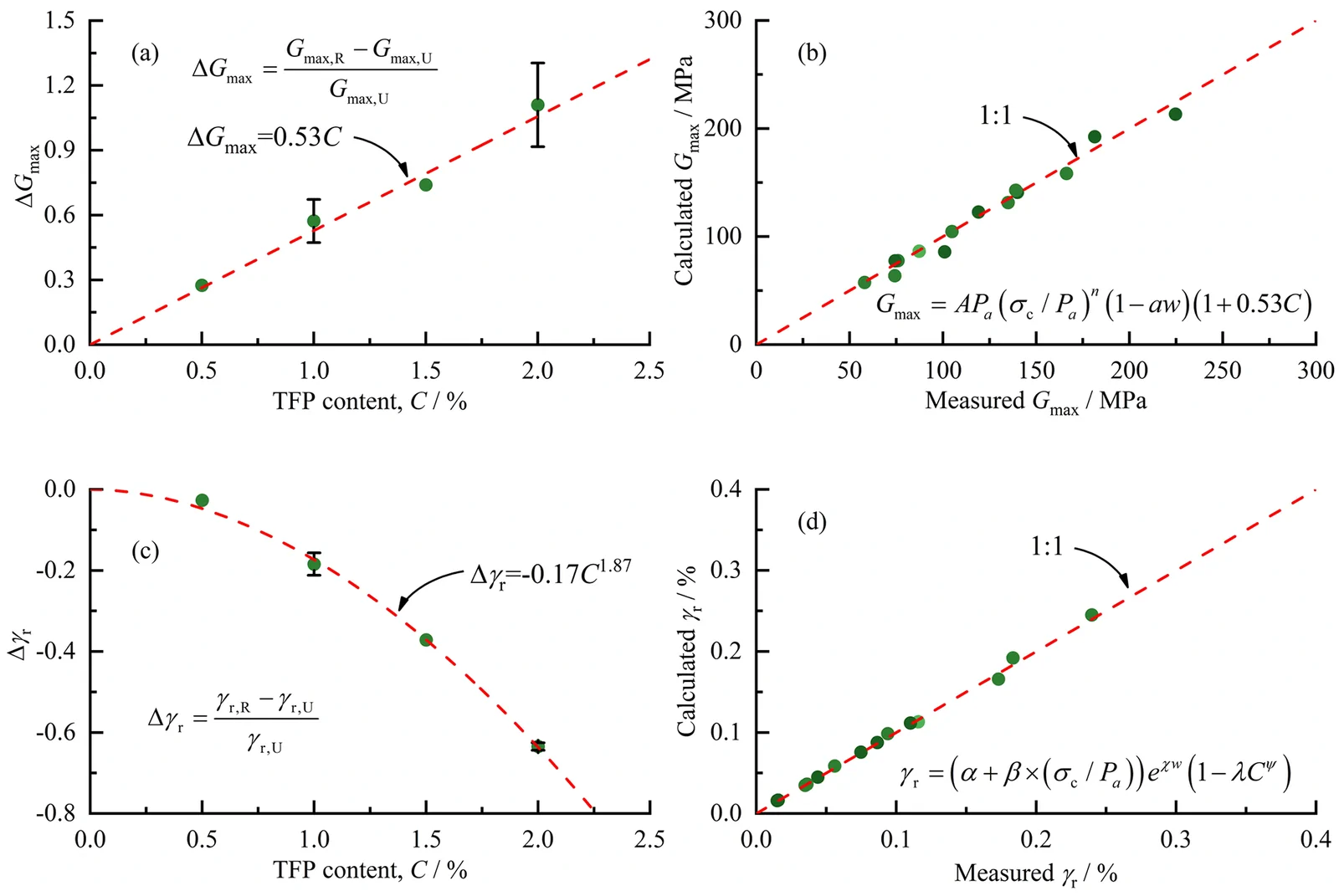
Modified models and performance considering TFP content: (a) relative increment of *G*_max_; (b) comparison between predicted and measured *G*_max_; (c) relative variation of *γ*_r_; (d) comparison between predicted and measured *γ*_r._


$$\begin{array}{c} G_{max}=AP_{a}{\left(\sigma_{\text{c}}/P_{a}\right)}^{n}\left(1-aw\right)\left(1+kC\right) \end{array}$$ (8)

where *A* is the empirical stiffness coefficient, *n* is the stress-dependent modulus exponent, and *a* is the water-dependent stiffness attenuation coefficient. The parameter *k* represents the relative stiffness enhancement coefficient associated with TFP content.

The comparison between measured and calculated *G*_max_ values (Fig 12b) shows that the data points are generally distributed close to the 1:1 identity line, indicating good agreement between the proposed model and the experimental results. This agreement supports the ability of the model to characterize the combined influences of applied confining pressure, water content, and TFP content on maximum dynamic shear modulus.

Regarding the reference shear strain *γ*_r_, Fig 12c demonstrates that its relative change rate ∆*γ*_r_ follows a power-law decay pattern with increasing TFP content. By incorporating a corresponding correction term, a comprehensive predictive model for *γ*_r_ was established:


$$\begin{array}{c} \gamma_{\text{r}}=\left(\alpha +\beta \times \left(\sigma_{\text{c}}/P_{a}\right)\right)e^{\chi w}\left(1-\lambda C^{\psi }\right) \end{array}$$ (9)

where *α* is the reference shear strain intercept, *β* represents the influence of applied confining pressure on *γ*_r_, and *χ* is the water-dependent evolution coefficient. The parameters *λ* and *ψ* characterize the magnitude and nonlinear dependence of the TFP-related correction to *γ*_r_, respectively.

Similarly, the comparison between measured and calculated *γ*_r_ values (Fig 12d) indicates that the revised model adequately describes the variation in reference shear strain associated with applied confining pressure, water content, and TFP content. Together, these results demonstrate that the proposed models provide a unified quantitative description of the small-strain dynamic shear characteristics of TFP-treated loess within the investigated experimental conditions.

The fitting performance of the revised models was further evaluated using the coefficient of determination (R^2^), root mean square error (RMSE), and mean absolute error (MAE) based on the 27 experimental conditions. For *G*_max_, the corresponding values were 0.99, 5.49 MPa, and 3.85 MPa, respectively. For *γ*_r_, the corresponding values were 0.99, 0.0056%, and 0.0035%, respectively. These quantitative metrics further characterize the agreement between the measured and calculated dynamic parameters.

## Microstructural characteristics of TFP reinforcement

Scanning electron microscopy (SEM) was employed to examine the microstructural features of untreated and TFP-treated loess (Fig 13). The untreated specimen (Fig 13a) exhibits a relatively open particle arrangement, with visible pores and localized interparticle contacts. After treatment with 1% TFP (Fig 13b), film-like features can be observed on particle surfaces and around interparticle contacts, suggesting possible modifications to particle surface morphology and local connections. At a TFP content of 2% (Fig 13c), more pronounced film-like coatings and filament-like features are observed around soil particles and adjacent contacts, indicating further changes in the local microstructural arrangement.

**Fig 13:**
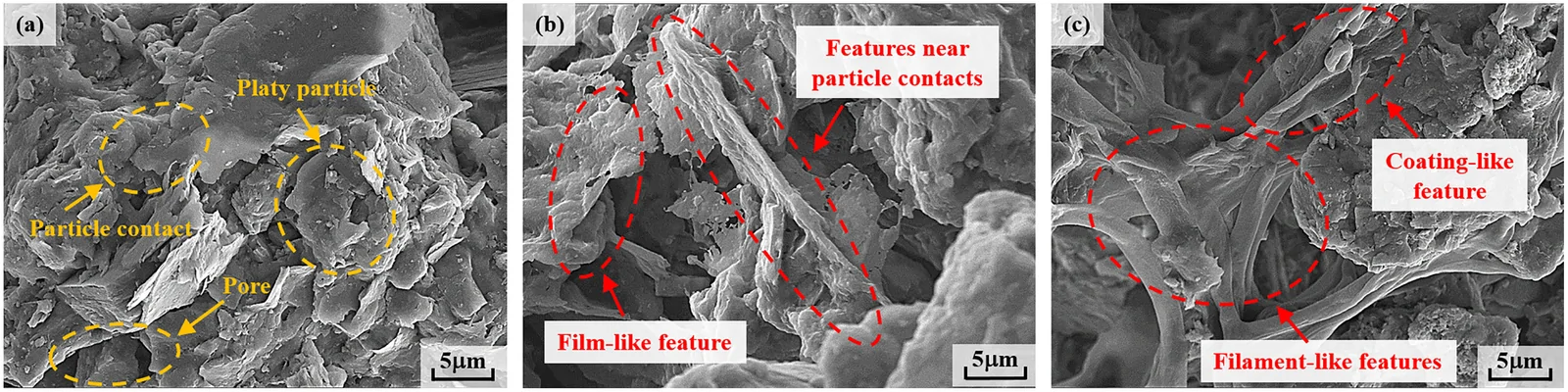
SEM micrographs of untreated and TFP-treated loess: (a) untreated loess (*C* = 0%); (b) TFP-treated loess (*C* = 1%); (c) TFP-treated loess (*C* = 2%).

These morphological features suggest that TFP treatment may modify interparticle contacts and connections, potentially contributing to the enhanced dynamic shear stiffness observed in treated specimens. This interpretation is consistent with the pronounced increase in the fitted parameter *G*_max,0_ shown in Fig 10b, while the moisture-dependent decay factor *a* exhibits only limited variation with TFP content. Together, the experimental and microstructural observations suggest that TFP reinforcement primarily improves the stiffness level of the soil skeleton under the investigated moisture conditions.

## Conclusions

A total of 27 sets of resonant column tests were conducted under varying applied confining pressures, water contents, and fungal polysaccharide (TFP) dosages. Coupled with scanning electron microscopy (SEM) characterization, this study aimed to elucidate the evolutionary mechanism of the dynamic properties of TFP-treated loess and to construct a predictive model considering the coupled effects of applied confining pressure, water content, and TFP dosage. The primary conclusions are as follows:

(1) Resonant column tests indicate that the dynamic shear modulus of both untreated and TFP-treated loess exhibits a characteristic nonlinear decay with increasing shear strain. The maximum shear modulus grows according to a power-law with applied confining pressure and attenuates linearly with water content, while the reference shear strain increases linearly with *σ*_c_ and escalates exponentially with *w*. Based on these findings, a unified predictive model incorporating multiple experimental variables was developed to quantitatively characterize the dynamic shear response of loess.
(2) TFP treatment substantially enhances the small-strain dynamic shear stiffness of loess across the investigated water contents. At a confining pressure of 150 kPa and a water content of 13%, *G*_max_ increases from 63.12 MPa for untreated loess to 139.93 MPa for 2% TFP-treated loess. Even at a water content of 20%, the corresponding *G*_max_ increases from 38.29 MPa to 74.38 MPa. The fitted parameter *G*_max,0_ increases markedly with TFP content, whereas the decay factor *a* exhibits only slight variation, indicating broadly consistent normalized moisture-dependent attenuation characteristics despite the pronounced improvement in absolute stiffness. In addition, the reinforcement effect becomes more pronounced at higher confining pressures, while changes in *γ*_r_ indicate that TFP treatment also influences the strain-dependent modulus reduction behavior.
(3) SEM observations reveal film-like coatings and filament-like features in TFP-treated loess, suggesting modifications to particle contacts and interparticle connections. These microstructural features may contribute to the enhanced dynamic shear stiffness of treated loess under different water contents. The combined experimental and microstructural findings provide a basis for understanding the stiffness enhancement associated with TFP reinforcement and its potential application to moisture-sensitive loess.
